# G26P[19] rotavirus A strain causing acute gastroenteritis in the American continent

**DOI:** 10.1590/0074-02760180344

**Published:** 2018-11-29

**Authors:** Mariela M Gómez, Marcelle FM da Silva, Eduardo M Volotão, Alexandre M Fialho, Christian S Mazzoco, Myrna S Rocha, José Paulo G Leite

**Affiliations:** 1 Fundação Oswaldo Cruz-Fiocruz, Instituto Oswaldo Cruz, Laboratório de Virologia Comparada e Ambiental, Rio de Janeiro, RJ, Brasil; 2 Hospital Municipal Jesus, Rio de Janeiro, RJ, Brasil

**Keywords:** rotavirus A, G26P(19), reassortment, rearrangement

## Abstract

In Brazil, the rotavirus A genotype G26 was first identified in suckling piglets, while the P[19] genotype has not been identified in any animal species so far. This report details the genetic characterisation of a G26P[19] RVA strain detected from an eight year-old child, vaccinated with Rotarix^®^, hospitalised with acute diarrhoeal disease in Rio de Janeiro in 2015. Most likely, the genome constellation (I5-R1-C1-M1-A8-N1-T1-E1-H1) observed in the G26P[19] Brazilian strain was a result of interspecies transmission events between humans and pigs. In addition, a rearrangement in the NSP5 gene was observed downstream of the 3’ non-coding region.

Rotaviruses are one of the leading causes of acute diarrhoeal disease (ADD) and dehydration in infants and young children throughout the world. To date, species A rotaviruses (RVA) that infect humans and animals have been classified into 36 G and 51 P genotypes based on differences in the VP7 and VP4 genes, respectively.^1^ RVA genotype G26 was reported for the first time in 2011 in pigs in Japan.^2^ Subsequent detections were reported in pigs in Kenya,^3^ in sewage in Italy,^4^ and recently in children with ADD in Nepal and Vietnam.^5^ ^,^ ^6^ In Brazil, G26 was reported in piglets with diarrhoea in combination with P[13] genotype from a sample collected in 2012 in Rio Grande do Sul state.^7^ Genotype P[19] is observed to commonly infect pigs but is considered sporadic in humans. After its first identification in pigs in China,^8^ this genotype was identified in human RVA strains from India,^9^ ^,^ ^10^ Thailand,^11^ and more recently from Italy.^4^ ^,^ ^12^ Previous studies reporting the detection of P[19] genotype in human and pig stool samples suggested that this genotype may have originated in pigs and introduced into the human population after events of reassortment between human and porcine RVA.^5^ ^,^ ^8^ ^,^ ^9^ ^,^ ^11^ ^,^ ^13^

The zoonotic potential of RVA animal strains is of significant concern, since genotypes commonly found in animals have been detected in sporadic and epidemic cases of ADD in human populations as observed previously in Brazil.^14^ ^,^ ^15^ ^,^ ^16^ ^,^ ^17^ ^,^ ^18^ ^,^ ^19^

In this study, the nearly complete genome of a human G26P[19] RVA strain from Brazil was characterised. This study is part of a project that encompasses the diagnosis, surveillance, and molecular epidemiology of viruses that cause ADD, approved by the Ethics Committee of Fiocruz (CEP: 311/06).

Stool was sampled from a child, who was eight years of age, diagnosed with ADD, hospitalised in a public hospital in Rio de Janeiro city, RJ, Brazil, 2015, who had received two doses of Rotarix^®^ in early infancy. Total RNA was extracted using QIAamp Viral RNA Mini Kit (Qiagen, Hilden, Germany), following the manufacturer’s instructions. Polyacrylamide gel electrophoresis (PAGE) analysis and silver staining of double-stranded (ds)RNA segments was performed as described by Pereira et al.^20^ Amplification was performed using OneStep real-time polymerase chain reaction (RT-PCR) Kit (Qiagen), following the manufacturer’s instructions. The PCR amplicons were purified using the QIAquick PCR purification kit (Qiagen). The strategy of primers and amplification conditions used were previously described by My et al.^5^ Amplicon sequencing was performed with ABI Prism Big Dye Terminator Cycle Sequencing Ready Reaction Kit and ABI Prism 3730 Genetic Analyzer (Applied Biosystems, Foster City, California, USA) by Genomic Platform of DNA sequencing PDTIS/Fiocruz. Nucleotide sequences of the 11 RVA encoding genes segments were deposited in GenBank under accession numbers: MG407645-55 (VP1-VP4, VP6, VP7, and NSP1-NSP5). Genotypes for each gene were identified using the RotaC online tool (URL: http://rotac.regatools.be)^21^ and nucleic acid sequences were compared with other RVA strains available at GenBank sequence database using the Basic Local Alignment Search Tool (BLAST) (URL: http://blast.ncbi.nlm.nih.gov/Blast.cgi). Phylogenetic analyses were performed using MEGA 7 software.^22^

**TABLE:** Genotype constellation comparison of Brazilian G26P[19] RVA (RVA/Human-wt/BRA/rj24598/2015/G26P[19]), and RVA reference strains of interest available at GenBank

Strain	Genotypes										
	VP7	VP4	VP6	VP1	VP2	VP3	NSP1	NSP2	NSP3	NSP4	NSP5
RVA/Human-wt/BRA/rj24598/2015/G26P[19]	G26	P[19]	I5	R1	C1	M1	A8	N1	T1	E1	H1
	990 bp	2328 bp	*1263 bp*	*3233 bp*	2701 bp	2508 bp	1494 bp	979 bp	1008 bp	723 bp	913 bp
RVA/Human-wt/VNM/30378/2009/G26P[19]	G26 **(98,0%)**	P[19] **(98,6%)**	I5 **(98,0%)**	R1 (85,7%)	C1 (90,2%)	M1 (88,3%)	A8 (79,3%)	N1 **(94,0%)**	T1 **(98,2%)**	E1 **(97,4%)**	H1 (92,5%)
RVA/Human-wt/NPL/07N1760/2007/G26P[19]	G26 (94,0%)	P[19] (93,8%)	I12	R1 (85,2%)	C1 (90,7%)	M1 (86,6%)	A8 (81,1%)	N1 (90,0%)	T1 (95,6%)	E1 (91,6 %)	H1 (93,7%)
RVA/Pig-wt/JPN/TJ4-1/2010/G26P[7]	G26	P[7]	X	X	X	X	X	X	X	X	X
RVA/Pig-wt/BRA/BRA381/2012/G26P[13]	G26 (93,8%)	P[13]	X	X	X	X	X	X	X	X	X
RVA/Pig-wt/IND/TRJRV/SW21/2014/G26P[X]	G26	X	X	X	X	X	X	X	X	X	X
RVA/Human-tc/THA/Mc323/1989/G9P[19]	G9	P[19] (93,5%)	I5	R1	C1	M1	A8	N1	T1	E1	H1
RVA/Pig-wt/BRA/ROTA09/2013/G3P[13]	G3	P[13]	I5 (89,4%)	R1 **(92,5%)**	C1 **(93,1%)**	M1 (91,2%)	A8 **(94,9%)**	N1 (87,5%)	T7	E1 (90,1%)	H1
RVA/Pig-wt/BRA/ROTA10/2013/G9P[23]	G9	P[23]	I5 (89,0%)	R1 (90,3%)	C1 (92,2%)	M1 **(91,3%)**	A8 (82,0%)	N1 (87,6%)	T1 (88,5%)	E1 (93,3%)	H1 (94,5%)
RVA/Pig-wt/BRA/ROTA17/2013/G5P[6]	G5	P[6]	I5 (91,0%)	R1 (91,5%)	C1 (93,0%)	M1 (89,7%)	A8 (91,4%)	N1 (87,8%)	T7	E1 (88,5%)	H1 **(98,3%)**

The length of the obtained sequences in this study are shown, the ones in italic means partial open reading frame (ORF) sequenced, otherwise the complete ORF was obtained. Percentage between brackets refers to the nucleotide identity value obtained after comparison with the rj24598 Brazilian strain, highest values are shown in bold. The “X” indicates the unavailability of sequence data in the GenBank. Gene segments of typical human Wa-like RVA origin are coloured green; blue is used to indicate gene segments of presumable porcine origin.

**Figure:**
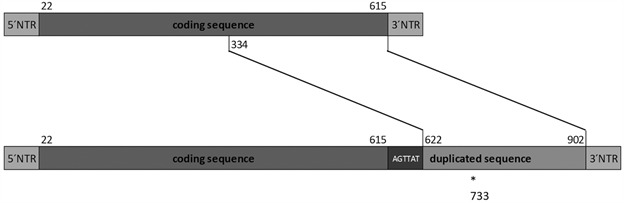
Schematic representation of the structure of normal and rearranged NSP5 gene of Brazilian G26P[19] RVA strain (RVA/Human-wt/BRA/rj24598/2015/G26P[19]). Insertion of “AGTTAT” sequence at position 616; duplication of a 281 bp long nucleotide segment; *deletion of a nucleotide at position 733. NTR: non-translated region. The numbering of the nucleotides is in respect to the expected complete gene segment based on RVA/Pig-tc/USA/Gottfried/1975/G4P[6] NSP5 prototype sequence, accession number GU199491.

PAGE analysis revealed a super-short RVA electropherotype [Supplementary data (Fig. 1)]. Following the observed electropherotype, analysis of the NSP5 sequenced amplicon revealed an unexpected length of 913 base pairs (5’UTR and 3’UTR regions were partially sequenced). Despite the NSP5 coding sequence being complete, it is followed by an insertion of “AGTTAT” sequence after the stop codon, a duplication of a 281 bp segment with nucleotide deletion at position 733, ending with 3’UTR (Figure). Rearrangements in the NSP5 gene segment have been reported previously in animal strains,^23^ ^,^ ^24^ ^,^ ^25^ ^,^ ^26^ and in two porcine-like human RVA strains.^27^ ^,^ ^28^ All rearrangements observed were present downstream of the stop codon, resulting in a normal gene translation, including the one described in this report. This protein plays an important role in several steps of viral replication (RNA binding, protein kinase, forms viroplasms with NSP2, and interaction with VP2 and NSP6).^29^ The rearrangement of sequence can directly influence viral fitness and its infectivity. However, further studies should be conducted in order to clarify this statement.

The Wa-like backbone genome constellation of the Brazilian G26P[19] strain was identical to verified Brazilian porcine RVA strains detected in 2013 from São Paulo and Mato Grosso states,^30^ as well as of a Vietnamese porcine-like human strain RVA/Human-wt/VNM/30378/2009/G26P[19]:^5^ I5-R1-C1-M1-A8-N1-T1-E1-H1. In addition, none of the G26P[19] RVA strains reported previously showed the rearrangement observed in the NSP5 genome segment of the Brazilian strain. Reassortment events among animal-human, animal-animal, and human-human RVA co-circulating strains contribute to the genetic heterogeneity observed in RVA.^29^ The introduction of an animal RVA segment into a human RVA strain is occasionally reported. The phylogenetic and sequence identity analysis of NSP1, NSP5, and VP1-3 revealed that the Brazilian G26P[19] strain analysed here was most closely related to the Brazilian RVA porcine strains [Table, Supplementary data (Figs 2-3)]. However, analysis of the remaining genes (NSP3-4, VP4, VP6-7) showed the highest nucleotide sequence identity to the Vietnamese 30378 (G26P[19]) strain and other porcine and/or porcine-like human RVA strains [Table, Supplementary data (Figs 2-3)]. Together, these results show that the Brazilian G26P[19] RVA strain may have arisen after several reassortment events occurring in porcine and/or human hosts, as suggested for previously described G26P[19] human RVA strains.^5^ ^,^ ^6^

This is the first report of genotype G26P[19] in the American continent, and also the first report of this particular genotype associated with ADD in a Brazilian child. While genotype G26 was already detected in pigs in Brazil,^7^ the genotype P[19] had not been detected in any animal species in Brazil till date. Analysis involving backbone genome sequencing of RVA strains is mostly performed in human samples. The close genetic relationship among the G26P [19] Brazilian RVA strain and porcine strains previously detected in Brazil shows the importance of sequencing the complete genome of animal RVA strains detected in Brazil. Notably, the absence of pre-existing population immunity against new strains might enable rapid distribution of new variants and its persistence in the population. Finally, this study highlights the importance of performing PAGE analysis in order to detect super-short electropherotypes and investigate the occurrence of rearrangement in porcine and porcine-like human RVA strains.
