# Supplementary material for: G26P[19] rotavirus A strain causing acute gastroenteritis in the American continent
Source: Mem Inst Oswaldo Cruz. 2018 Nov 29;113(12):e180344. doi: 10.1590/0074-02760180344 (PMC6254902; doi:10.1590/0074-02760180344)
Supplement: Supplementary file 1 [file 1678-8060-mioc-113-12-e180344-s.pdf]

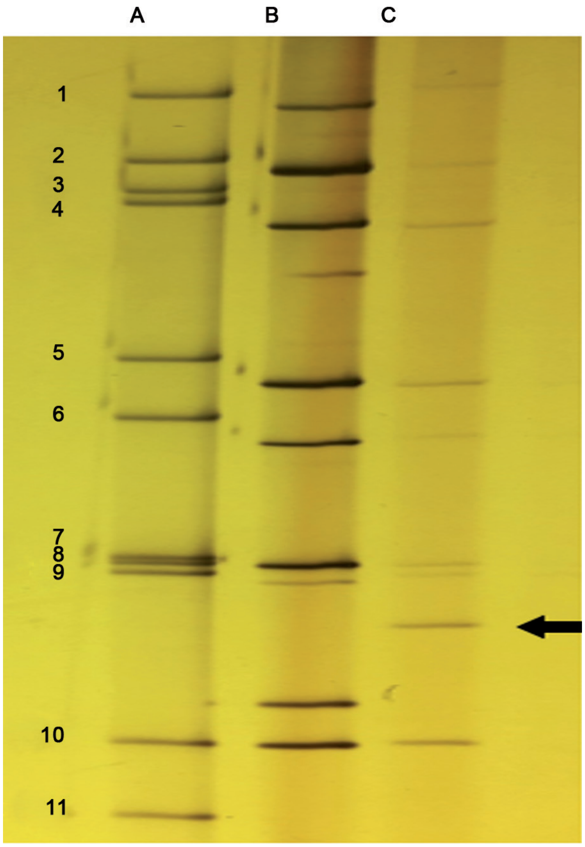

Fig. 1: electropherotype profile of RVA strains. SA11 (A) strain possesses a long RNA pattern, sample 29555 (B) possesses a short RNA pattern, and rj24598 (C) possesses a super-short pattern. Note: The lowest migration rate of gene segment 11 of rj24598 strain is indicated by an arrow. The positions of RNA segments 1 through 11 of strain SA11 are indicated.

cc BY

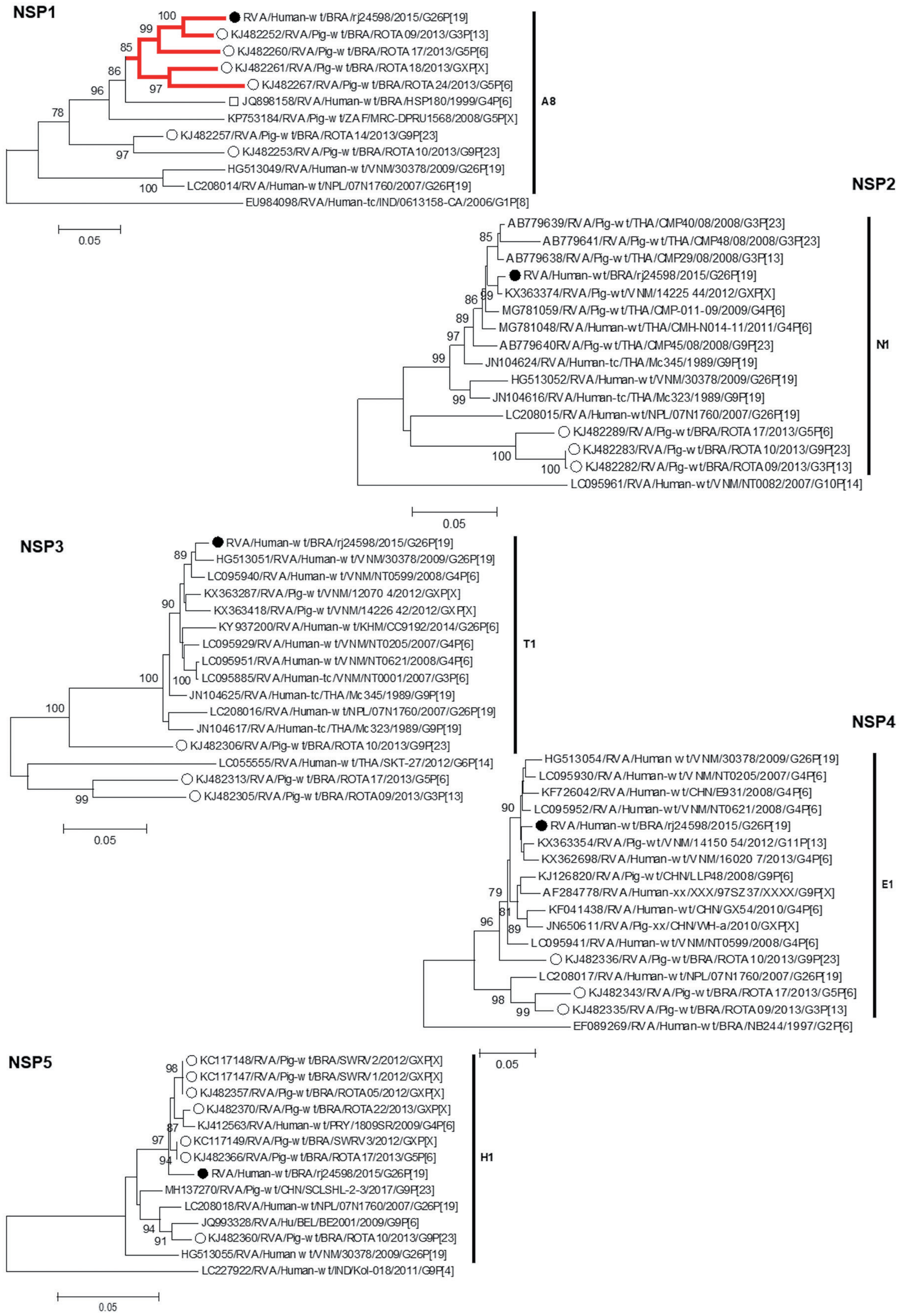

Fig. 2: Neighbour-Joining phylogenetic trees based on non-structural protein coding genes: NSP1-5. The Brazilian G26P[19] RVA strain analysed in this study is marked with a filled circle; porcine strains detected in Brazil are marked with an empty circle; human strains detected in Brazil are marked with an empty square. In cases where the Brazilian G26P[19] RVA strain formed a cluster with porcine RVA Brazilian strains, the branches are shown in red colour. Bootstrap values (1000 replicates) above 70 are shown in the corresponding nodes. The genotype is shown on the right side of each tree. The scale bar at the bottom represents 0.05 substitutions per nucleotide position (nt.subst./site).

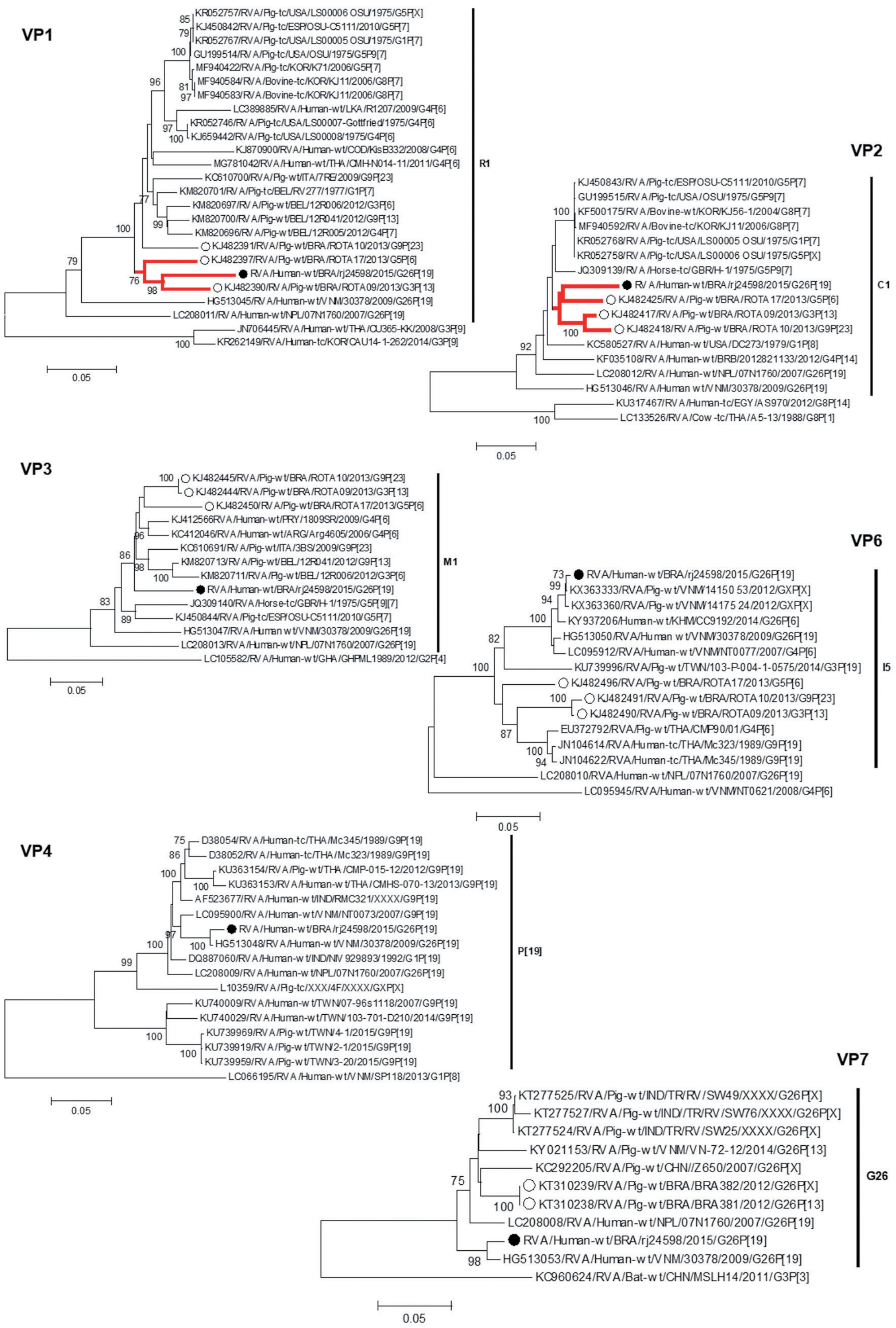

Fig. 3: Neighbour-Joining phylogenetic trees based on structural protein coding genes: VP1-4, VP6-7. The Brazilian G26P[19] RVA strain analysed in this study is marked with a filled circle; porcine strains detected in Brazil are marked with an empty circle. In cases where the Brazilian G26P[19] RVA strain formed a cluster with porcine RVA Brazilian strains, the branches are shown in red colour. Bootstrap values (1000 replicates) above 70 are shown in the corresponding nodes. The genotype is shown on the right side of each tree. The scale bar at the bottom represents 0.05 substitutions per nucleotide position (nt.subst./site).
